# Reliability of Online Surveys in Investigating Perceptions and Impressions of Faces

**DOI:** 10.3389/fpsyg.2021.733405

**Published:** 2021-09-22

**Authors:** Naoyasu Hirao, Koyo Koizumi, Hanako Ikeda, Hideki Ohira

**Affiliations:** ^1^Shiseido Global Innovation Center, Yokohama-Shi, Japan; ^2^Department of Psychology, Graduate School of Informatics, Nagoya University, Nagoya, Japan

**Keywords:** face, impression, survey, online, reliability – reproducibility of results

## Abstract

Online experimental methods are used in psychological studies investigating the perceptions and impressions of facial photographs, even without substantial evidence supporting their reliability and validity. Although, the quality of visual stimuli is more difficult to control remotely, the methods might allow us to obtain a large amount of data. Then the statistical analysis of a larger volume of data may reduce errors and suggest significant difference in the stimuli. Therefore, we analyzed the reliability and validity of online surveys in investigating the perceptions (shine, red, and dark) and impressions (attractiveness, trustworthy, and so on) of facial photographs created from averaged faces with skin tones modified using computer graphics (CG). In this study, we conducted online (Online1) and laboratory experiments with well-controlled conditions (Control). For each experiment, 50 participants (men and women in Japan, age: 20–59years) completed the same questionnaire regarding their impressions of the same 28 CG facial photographs. The results showed significant correlations between the two experiments for all 19 items in the questionnaire. SD in the Online1 compared to the Control from the stimuli and individual differences were 56–84 and 88–104% in each questionnaire items, respectively. Moreover, the rates of mismatching perceptual evaluations to the corresponding physical features demonstrated in the photographs were 4.9–9.7% on average in an additional online survey of another 2,000 participants (Online2). These results suggest that online surveys can be applied to experiments to investigate impressions from CG facial photographs instead of general laboratory experiment by obtaining an appropriate number of participants to offset larger statistical errors that may result from the increased noise in the data from conducting the experiment online.

## Introduction

In studies of perception and impressions regarding faces, facial photographs have been used as stimuli in experiments that investigate visual illusions in facial perception (Baker et al., 2007; Matsushita et al., 2015) and the relationship between specific facial features and impressions of a person (Fink et al., 2006; Samson et al., 2010; Jaeger et al., 2018). In studies conducted in a laboratory, facial photographs are presented as printed media or on computer displays with controlled color and brightness and under well-controlled lighting conditions. This is because appropriate visual conditions of the stimuli are desirable for the participants to properly perceive their differences, reducing the noise in the evaluation.

With the widespread use of the Internet, online surveys allow participants to take part in research despite not being physically present in a laboratory. This is especially important advantage of online surveys during the current coronavirus pandemic as of course online surveys cut the risk of transmission of infectious disease. In online settings, the cost of recruiting participants and providing a suitable research location can be used to acquire a larger number of participants instead. Although, there is a potential disadvantage in this approach due to the increase the error in the evaluation of each stimulus (due to less control over visual stimuli among participants and the subsequent increase in individual differences.), the larger data sample may be an advantage in detecting evaluation differences within the stimuli because the SE is inversely proportional to the square of the sample size.

Some reports have investigated the reliability and validity of online surveys compared to their paper-based counterparts (Carrascosa et al., 2011) or compared to existing laboratory research (Crump et al., 2013) and the limitation of online versions (Alessi and Martin, 2010; Ball, 2019). For example, Crump et al. (2013) found that a variety of commonly used tasks performed online produced results broadly consistent with laboratory results. However, the validity and reliability of online surveys for investigating impressions from facial photographs remains unknown. This is important because, unlike linguistic stimuli, the subjective impression of a face photograph may change depending on conditions under which the photograph is viewed. For example, the radiance of a face can affect the impression of it (Ikeda et al., 2021). The radiance of the face can be affected by the room conditions and display screen conditions, neither of which can be controlled easily in an online experiment.

Therefore, we first investigated the difference between data from an online experiment (henceforth referred to as “Online1”) and data from a typical experiment in a laboratory with well-controlled stimuli and experimental conditions, including lighting (henceforth referred to as “Control”). Specifically, the current study aimed to investigate the validity and reliability of an online survey of perception of and impressions formed by faces. We also attempted to calculate the necessary number of participants for an online survey if the same level of statistic reliability was required. In both experiments, 50 participants evaluated the same 28 computer graphics (CG) stimuli faces that varied by age, sex, and skin features. The faces were evaluated in 19 items of the questionnaire, which assessed the perceptual features and impressions using a five-point Likert scale. Then, the validity and reliability of the online surveys was evaluated by examining the correlations between the values evaluated in the two experiments. If the correlations of the data in online survey to a typical experiment in a laboratory with well-controlled stimuli are statistically significant, the online method could be regarded as valid because it suggests that online survey can provide the same data of evaluations. At the same time, the level of the contribution rates from the correlation analysis presented by the *R*^2^ scores could suggest the reliability, which represents the stability of the results. Reliability in experimental data is not only dependent on the procedure to obtain the data but also the sample size because it is related to the statistical error. Therefore, based on the observed correlation coefficients, we can calculate the appropriate number of participants in a future online survey to maintain the same level of reliability based on the difference in the error due to the stimuli and the individual differences.

In addition, we also investigated the rates of mismatches in the physical features of the stimuli and the perceptual items. By examining these rates, it may be possible to find a limitation in the perception of the stimuli, which is dependent on the quality of the visual stimuli. As the number of participants in the Online1 (*n*=50), was not expected to be sufficient for this purpose, we conducted an additional online experiment with 2,000 participants, constituting “Online2.” Online2 used the same methods as the original online experiment. We compared the rates of mismatches for participants by age and sex. If the rates were high in a certain group of participants and in certain items in the questionnaire, this would suggest a limitation of online experimentation of this type.

## Materials and Methods

### Participants

A total of 100 healthy men and women living in Japan [50 men and 50 women, mean age: 39.9years (11.1 SD)] participated in Online1 and Control; a separate group of 2,000 healthy men and women living in Japan [1,000 men and 1,000 women, mean age: 40.0years (11.0 SD)] participated in Online2. Each participant indicated no optical disorders by self-report before the experiment and owned a personal computer or a tablet computer, excluding mobile phones with a small display, to participate in this experiment in Online1 and Online2. Each participant provided informed consent to participate in the study. The Research Ethics Committee of the Shiseido Global Innovation Center approved this study, and all methods were conducted following approved guidelines.

### Stimuli

**Figure 1:**
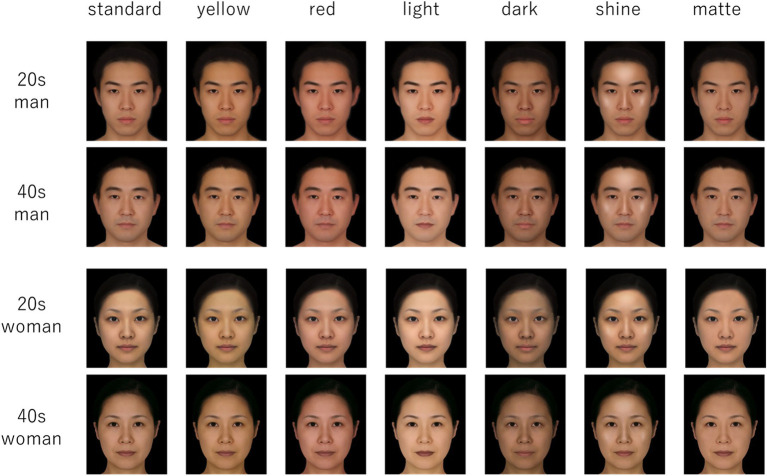
Facial photographs used in both experiments.

Twenty-eight CG facial photographs were used as visual stimuli. These were divided into four groups according to age (20s vs. 40s) and sex (male vs. female). For each group of stimuli, there were seven photographs with different skin features (original averaged face, darker skin, brighter skin, redder skin, yellower skin, shiny skin, and matte skin). From eight facial photographs, four original averaged faces were created: Japanese men in their 20s, Japanese men in their 40s, Japanese women in their 20s, and Japanese women in their 40s. The darker and brighter faces were created from the original averaged face by making the skin tone darker or brighter by +2 or −2 SDs from L* on the cheek, based on the distribution of skin color from a previous experiment by the authors (unpublished). The red and yellow images were also created by adjusting the color of the original face to +2 SD or −2 SDs based on the a* and b* distributions for red and yellow, respectively, from the same study. The shiny and matte images were created by a professional CG creator who altered the contrast of the skin color. Photoshop CS4 (version 11; Adobe Inc., 2008) was used in the process of creating the stimuli (Figure 1).

### Questionnaire

Questionnaire items included three perceptual features that used bipolar scales (dark–bright, red–yellow, and shiny–matte) and 16 items regarding impressions of the face measured using monopole scales (trustworthy, honest, reliable, confident, attractive, likable, healthy, youthful, clean-cut, want to be, cool, energetic, polished, beautiful, good at work, and unisex). We selected six items (trustworthy, honest, reliable, confident, attractive, and likable) from a previous study as the basic impressions from appearance (Jaeger et al., 2018) and added the other 10 items. All questionnaire items used a five-point Likert scale, with scores ranging from −2 to +2. For example, the dark–bright dipole scale appeared as follows (translated from the original Japanese): +2, “match to dark”; +1, “a slight match to dark”; 0, neutral; −1, “match to bright”; and −2, “a slight match to bright.” The monopole scale categories appeared as follows: +2, “I think so very much”; +1, “I think so”; 0, neutral; −1, “I do not think so”; and −2, “I do not think so very much.”

### Procedure

This study included an online experiment (Online1) and an experiment using printed photographs in a laboratory (Control). In Online1, 50 participants looked at the stimuli on their personal computers or tablets and filled in the questionnaire at home. An in-house platform for online experiments was used. In the control condition, under well-controlled lighting, the other 50 participants were in the laboratory, looked at the same stimuli printed on paper (210×297mm), and filled in the same questionnaire. In both of the experiments, participants filled in the list of three perceptual questionnaire items and the other 16 items of impressions after looking at one of visual stimuli at their own pace in a block. They repeated 28 blocks for each visual stimulus assigned to each of them. The orders of stimuli and the questionnaire items in each of perceptions and impressions were also randomized.

After the two experiments, we conducted an additional online experiment with additional participants using the same method as Online1, the 28 original stimuli, and the three perceptional items of the questionnaire; this constituted Online2. In the three experiments, the participants were divided into two groups. The first group evaluated 14 stimuli portraying males and females in their 20s, while the second group evaluated 14 stimuli portraying males and females in their 40s.

### Statistical Analysis

The average scores for both Online1 and Control groups were calculated for each item. To investigate the validity of the Online1, the tests of significance were applied to Pearson’s product-moment correlation coefficients for the 28 scores between the two experiments, with a criterion of *α*=0.05. Furthermore, the ratios of SDs of Online1 vs. Control conditions were calculated both for the differences in the stimuli (*a*=ratio of the SDs of the scores averaged for the 28 stimuli for the 50 participants) and the individual participants (*b*=ratio of the averaged SDs of 28 stimuli for the 50 participants) to calculate the ratio of the required sample size in a future online experiment and match the effect sizes in the control using the formula b^2^/a^2^.

As for the analysis of Online2, the calculated total rates of mismatched answers between the physical features (e.g., the rate calculated from the number of participants who evaluated the CG face of dark skin brighter compared to the brighter skin CG face) were averaged for the four types of stimuli (CG facial photographs: darker skin vs. brighter skin, red skin vs. yellow skin, and shiny skin vs. matte skin). The values suggest how precisely participants perceive the optical features in the visual stimuli. The perceptual evaluations associated with items in the questionnaire (dark–bright, red–yellow, and shiny–matte) for each age group (20s, 30s, 40s, and 50s) and participant gender groups (male and female) were also calculated. The difference in the ratios among the groups was examined using the chi-square test (*α*=0.05).

## Results

### Testing Validity

**Figure 2:**
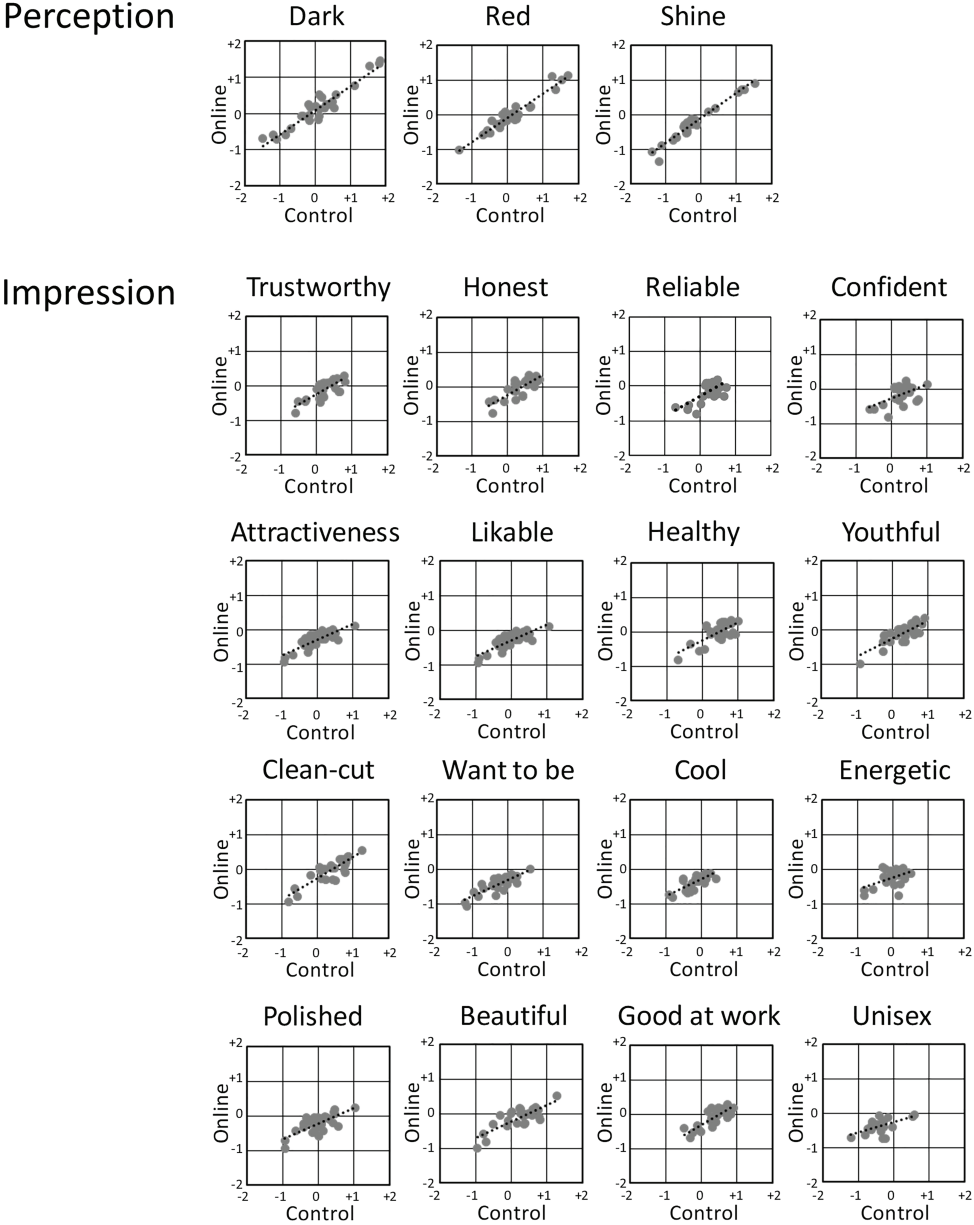
Correlation between the Online1 and Control averaged scores for each questionnaire item for 28 stimuli.

The validity of Online1 was tested by correlating the results with that of Control. The correlations of the tested items between Online1 and Control were statistically significant for all the 19 items (*p*<0.01; Table 1; Figure 2), suggesting that Online1 result was valid.

**Table 1:** Correlations and differences of errors for each item for the Online1 and Control conditions.

Questionnaire items		Correlation between online and control	SD calculated from averaged scores of 28 stimuli			Averaged score of 28 stimuli SDs of 50 participants		
		*R* ^2^	Online1	Control	Ratio (O/C)	Online1	Control	Ratio (O/C)
Perception	Shine	0.951^*^	0.523	0.710	73.7%	0.884	0.945	93.6%
	Red	0.931^*^	0.506	0.701	72.1%	0.819	0.910	90.0%
	Dark	0.911^*^	0.568	0.808	70.2%	0.895	0.949	94.3%
Impression	Clean-cut	0.742^*^	0.345	0.489	70.5%	0.859	0.857	100.2%
	Like	0.731^*^	0.265	0.463	57.2%	0.854	0.965	88.5%
	Want to be	0.707^*^	0.249	0.446	55.7%	0.908	0.980	92.7%
	Attractiveness	0.678^*^	0.250	0.444	56.4%	0.847	0.926	91.5%
	Good at work	0.647^*^	0.263	0.349	75.4%	0.849	0.813	104.4%
	Beautiful	0.646^*^	0.309	0.520	59.4%	0.894	0.919	97.2%
	Honest	0.637^*^	0.274	0.376	73.0%	0.850	0.815	104.2%
	Trustworthy	0.615^*^	0.269	0.359	74.9%	0.827	0.790	104.8%
	Youthful	0.614^*^	0.276	0.405	68.0%	0.897	0.927	96.7%
	Cool	0.554^*^	0.192	0.293	65.6%	0.843	0.940	89.7%
	Polished	0.532^*^	0.267	0.437	61.1%	0.836	0.891	93.8%
	Healthy	0.496^*^	0.277	0.381	72.7%	0.893	0.978	91.3%
	Reliable	0.483^*^	0.264	0.314	84.1%	0.845	0.888	95.2%
	Unisex	0.326^*^	0.185	0.351	52.7%	0.867	0.958	90.5%
	Confident	0.289^*^	0.259	0.343	75.7%	0.840	0.909	92.3%
	Energetic	0.240^*^	0.226	0.325	69.4%	0.836	1.014	82.4%

* *p*<0.01.

O/C: ratios of the Online1 to the Control.

### Estimating Number of Participants Needed for Reliability

The ratio of the estimated participants in the online survey compared to the controlled experiments to maintain the same level of error was calculated by the formula b^2^/a^2^ for each questionnaire item. a was the ratios of SDs of the averaged scores for the 28 stimuli for the 50 participants for Online1 compared to Control. These ratios ranged from 56 to 84%. All 19 questionnaire items had significantly smaller SDs in Online1 than Control in the chi-square test (*χ*^2^=19.0, df=1, *p*<0.001). b was the ratio of average SDs of the 28 stimuli for the 50 participants for Online1 compared to Control. These ratios ranged from 88 to 104%. Also, the number of questionnaire items with a smaller average SD in the Online1 was 15 of 19, which was statistically significant in the chi-square test (*χ*^2^=6.37, df=1, *p*=0.012; Table 1). The values of b^2^/a^2^ ranged from 1.3 to 2.6 for each item, suggesting that online experiments may need 1.3–2.6 times the number of participants to obtain a comparable result as a laboratory experiment.

### Mismatched Answers Between the Physical Features

Moreover, we investigated the differences in the mismatch rates of perceptions within the participant groups to investigate whether there are any limitations in a specific category of participants. The values of the mismatch rates suggest the rates of participants who did not perceive and evaluate the physical features of stimuli precisely. In the results from Online2, the rates of mismatch between the physical features (darker skin vs. brighter skin, red skin vs. yellow skin, and shiny skin vs. matte skin) and the evaluations (dark–bright, red–yellow, and shiny–matte) were 4.9, 5.9, and 9.3%, respectively (Table 2). The differences within the four age groups regarding the mismatch rate were not significant according to the chi-square tests for the three questionnaire items. However, the difference between the male and female participants for the “shiny skin vs. matte skin” item was statistically significant for the chi-square test (*χ*^2^=12.10, df=1, *p*<0.001; Table 2). The difference may suggest a limitation in the reliability of the data in specific segments of the participants.

**Table 2:** The rates of inversely matching physical features to perceptual evaluations in the Online2.

Matching of stimuli features and answers		Participant characteristics						
	Age and sex groups	20s	30s	40s	50s	Male	Female	Total
	Number of participants	500	500	500	500	1,000	1,000	2,000
Dark vs. Bright	Did not evaluate the dark item as darker rather than brighter	5.0%	5.6%	3.9%	5.2%	5.5%	4.4%	4.9%
	Evaluated the image as equally dark and bright	13.2%	13.8%	11.6%	15.2%	14.3%	12.6%	13.5%
	Evaluated the dark item as darker rather than brighter	81.8%	80.6%	84.5%	79.6%	80.2%	83.1%	81.6%
Red vs. Yellow	Did not evaluate the red item as redder than yellower	5.6%	6.4%	5.5%	6.0%	6.5%	5.3%	5.9%
	Evaluated the image as equally red and yellow	20.3%	21.5%	21.0%	23.4%	22.9%	20.3%	21.6%
	Evaluated the red item as redder than the yellower	74.1%	72.1%	73.5%	70.6%	70.7%	74.5%	72.6%
Shiny vs. Matte	Did not evaluate the shine item as shiner than matter	10.0%	9.6%	9.5%	8.0%	11.0%	7.6%	9.3%
	Evaluated the image as equally shiny and matte	20.8%	25.1%	23.7%	26.0%	27.5%	20.3%	23.9%
	Evaluated the shine item shiner than matter	69.2%	65.3%	66.8%	66.0%	61.6%	72.1%	66.8%

## Discussion

In the current study, we investigated the reliability and validity of an online survey of perceptions and impressions of faces, by examining correlations between face evaluations obtained in an online survey (Online1) with face evaluations obtained in a well-controlled laboratory setting (Control), and estimating the number of participants needed for to maintain reliability and examining mismatch rates of perceptions in a larger online survey (Online2). We found positive correlations between Online1 and control experimental data for all the questionnaire items, suggesting that online survey is valid, but that the number of participants needed is 1.3–2.6 times that of a laboratory survey. In addition, there were generally low rates of mismatch.

The positive correlations suggest that the online survey and the general laboratory survey produced comparable results. The values of *R*^2^ between the Online1 and Control suggested moderate or low correlations for the items regarding impression (0.240–0.742) compared to items regarding perceptions (0.911–0.951). Nonetheless, because values of *R*^2^ are dependent on SDs in the differences of the stimuli, which, even in the general survey, were smaller for the items of impressions based on the perceptions in the information processing than the evaluation of the perceptions themselves. These results support the validity of online surveys.

Regarding reliability, the differences in the average scores of the stimuli were smaller in the online than in the control group for all items. This suggests that the online survey requires a larger sample size than the traditional, well-controlled lab-based survey. The required online sample size was estimated to be 1.3–2.6 times larger compared to the control based on the difference of errors within the two methods from the differences of stimuli and individual differences; these values may vary based on additional data and may depend on specific methods. Namely, differences in the stimuli were difficult to observe because of difficulty controlling the quality of visual stimuli in an online survey; however, this can be partially overcome by increasing the sample size.

The mismatching rates found in Online2 were less than 10% suggesting that participants had no difficulty in perceiving the online face stimuli. However, the mismatch rate between men and women was different for the item regarding shiny–matte skin. It is unclear however, if this mismatch rate difference indicates a genuine sex based difference in perception of the shiny/matte appearance of online face photographs or simply a difference in understanding of the meanings of the words in the participant segments, which may be the case in the original Japanese word used for shiny.

In other words, the current study overall supports the use of online surveys for testing the perception and impressions of face stimuli. However, it should be noted, that differences in the results of online and laboratory surveys may arise depending on the method used to recruit participants and several other factors, including their subsequent comprehension of the survey, possible deception in their answers, and communication with the researchers (Zhou and Fishbach, 2016). Online surveys using visual stimuli can also be limited by the characteristics of devices used by participants; device type should thus be considered carefully, especially in experiments using a between-subject design. Although, the types of devices used were limited to personal and tablet computers in the current surveys, the differences between groups in the scores might also be influenced by the bias of the device types used within specific consumer segments.

In addition, in the current study all participants were Japanese and evaluated CG Japanese faces. Though, we found no evidence that the different skin colors were affected differently by the lack of control over e.g., room brightness in the online tests, further research is needed to determine if this can be generalized to more diverse groups of participants and face stimuli.

The results in the current study suggest that online surveys can be applied to experiments to investigate impressions from CG facial photographs instead of general laboratory experiment, with the caveat that the number of participants should be increased. Although, there are some potential limitations in online surveys, they may potentially play a significant role as a substitute for laboratory experiments regarding the perception of faces. The benefits may outweigh the potential limitations especially during the current pandemic. The current study adds to the growing list of reports in behavioral science (Chesney et al., 2009; Crump et al., 2013; Hergueux and Jacquemet, 2015) and psychophysics (Semmelmann and Weigelt, 2017) that support the use of online experimental methods. Online surveys appear to be an effective method for investigating the perception and impression of faces using photographs.

## Data Availability Statement

The datasets presented in this article are not readily available due to confidentiality agreements with the participants; the data in this study are available only at the Shiseido Global Innovation Center. Requests to access the datasets should be directed to naoyasu.hirao@shiseido.com.

## Ethics Statement

The studies involving human participants were reviewed and approved by The Research Ethics Committee of the Shiseido Global Innovation Center. The patients/participants provided their written informed consent to participate in this study.

## Author Contributions

NH, KK, HI, and HO contributed to conception design of the study. NH and KK performed the statistical analysis. NH wrote the first draft of the manuscript by the support of HI. HO checked and revised the manuscript as the senior author. All authors contributed to the article and approved the submitted version.

## Funding

The present work was funded by the Shiseido Global Innovation Center.

## Conflict of Interest

The authors declare that the research was conducted in the absence of any commercial or financial relationships that could be construed as a potential conflict of interest.

## Publisher’s Note

All claims expressed in this article are solely those of the authors and do not necessarily represent those of their affiliated organizations, or those of the publisher, the editors and the reviewers. Any product that may be evaluated in this article, or claim that may be made by its manufacturer, is not guaranteed or endorsed by the publisher.
